# Incidence and Predictors of Infections and All-Cause Death in Patients with Cardiac Implantable Electronic Devices: The Italian Nationwide RI-AIAC Registry

**DOI:** 10.3390/jpm12010091

**Published:** 2022-01-11

**Authors:** Giuseppe Boriani, Marco Proietti, Matteo Bertini, Igor Diemberger, Pietro Palmisano, Stefano Baccarini, Francesco Biscione, Nicola Bottoni, Antonio Ciccaglioni, Alessandro Dal Monte, Franco Alberto Ferrari, Saverio Iacopino, Marcello Piacenti, Daniele Porcelli, Stefano Sangiorgio, Luca Santini, Michele Malagù, Giuseppe Stabile, Jacopo Francesco Imberti, Davide Caruso, Massimo Zoni-Berisso, Roberto De Ponti, Renato Pietro Ricci, on behalf of RI-AIAC Registry Investigators

**Affiliations:** 1 Cardiology Division, Department of Biomedical, Metabolic and Neural Sciences, University of Modena and Reggio Emilia, Policlinico di Modena, 41125 Modena, Italy; jacopo.imberti@hotmail.it; 2 Geriatric Unit, IRCCS Istituti Clinici Scientifici Maugeri, 20138 Milan, Italy; marco.proietti@unimi.it; 3 Department of Clinical Sciences and Community Health, University of Milan, 20122 Milan, Italy; 4 Liverpool Centre for Cardiovascular Science, University of Liverpool and Liverpool Heart & Chest Hospital, Liverpool L7 3FA, UK; 5 Cardiological Center, University of Ferrara, 44124 Ferrara, Italy; doc.matber@gmail.com (M.B.); mlgmhl1@unife.it (M.M.); 6 Department of Experimental, Diagnostic and Specialty Medicine, Institute of Cardiology, University of Bologna, Policlinico S. Orsola-Malpighi, 40138 Bologna, Italy; igor.diemberger@gmail.com; 7 Cardiology Unit, ‘Card. Giovanni Panico’ Hospital, 73039 Tricase, Italy; dr.palmisano@libero.it; 8 Cardiology Unit, Emergency Department, Fidenza Hospital, 43036 Fidenza, Italy; stefano.baccarini@gmail.com; 9 Cardiology Unit, Santo Spirito Hospital, 00193 Rome, Italy; francesco.biscione@poste.it; 10 Santa Maria Nuova Hospital, 42123 Reggio Emilia, Italy; nicola.bottoni@asmn.re.it; 11 Department of Cardiovascular Sciences, Sapienza-University of Rome, 00161 Rome, Italy; Antonio.Ciccaglioni@uniroma1.it; 12 Cardiology Unit, Santa Maria delle Croci Hospital, 48121 Ravenna, Italy; dalmo_it@yahoo.it; 13 Cardiology Unit, Rho Hospital, 20017 Rho, Italy; fa.ferrari@virgilio.it; 14 Electrophysiology Unit, Maria Cecilia Hospital, 48033 Cotignola, Italy; iacopino@iol.it; 15 Fondazione Toscana ‘Gabriele Monasterio’, 56124 Pisa, Italy; piacenti@ftgm.it; 16 Arrhythmology Unit, Cardiology Department, S. Giovanni Calibita Fatebenefratelli Hospital, 00186 Rome, Italy; daniele.porcelli@hotmail.it; 17 Electrophysiology Unit, Fatebenefratelli Hospital, 20121 Milan, Italy; dott.sangiorgiostefano@gmail.com; 18 Department of Cardiology, Ospedale GB Grassi, 00122 Ostia, Italy; lsantini@alice.it; 19 Department of Cardiology, Clinica Montevergine, 83013 Mercogliano, Italy; gmrstabile@tin.it; 20 Padre Antero Micone Hospital, ASL 3 “Genovese”, 16153 Genova, Italy; davidecaruso2000@yahoo.it (D.C.); massimo.zoniberisso@libero.it (M.Z.-B.); 21 Cardiovascular Department, Circolo Hospital, University of Insubria, 21100 Varese, Italy; roberto.deponti@uninsubria.it; 22 CardioArrhythmology Center, 00152 Rome, Italy; renatopietroricci@gmail.com

**Keywords:** cardiac implantable electronic device, implantable defibrillator, pacemaker, infection, outcome

## Abstract

Background: The incidence of infections associated with cardiac implantable electronic devices (CIEDs) and patient outcomes are not fully known. Aim: To provide a contemporary assessment of the risk of CIEDs infection and associated clinical outcomes. Methods: In Italy, 18 centres enrolled all consecutive patients undergoing a CIED procedure and entered a 12-months follow-up. CIED infections, as well as a composite clinical event of infection or all-cause death were recorded. Results: A total of 2675 patients (64.3% male, age 78 (70–84)) were enrolled. During follow up 28 (1.1%) CIED infections and 132 (5%) deaths, with 152 (5.7%) composite clinical events were observed. At a multivariate analysis, the type of procedure (revision/upgrading/reimplantation) (OR: 4.08, 95% CI: 1.38–12.08) and diabetes (OR: 2.22, 95% CI: 1.02–4.84) were found as main clinical factors associated to CIED infection. Both the PADIT score and the RI-AIAC Infection score were significantly associated with CIED infections, with the RI-AIAC infection score showing the strongest association (OR: 2.38, 95% CI: 1.60–3.55 for each point), with a c-index = 0.64 (0.52–0.75), *p* = 0.015. Regarding the occurrence of composite clinical events, the Kolek score, the Shariff score and the RI-AIAC Event score all predicted the outcome, with an AUC for the RI-AIAC Event score equal to 0.67 (0.63−0.71) *p* < 0.001. Conclusions: In this Italian nationwide cohort of patients, while the incidence of CIED infections was substantially low, the rate of the composite clinical outcome of infection or all-cause death was quite high and associated with several clinical factors depicting a more impaired clinical status.

## 1. Introduction

In recent years, there has been a progressively increasing use of cardiac implantable electronic devices (CIEDs), both due to the constant technological advance and to the evidence showing a significant effectiveness in improving patients’ quality of life and long-term survival [1,2]. Over a large spectrum of clinical indications, permanent pacemakers (PMs), implantable cardioverter-defibrillators (ICDs) and cardiac resynchronization therapy (CRT-D (with defibrillator) and CRT-P (without defibrillator)) use have all progressively increased in the last 15 years [3,4].

In patients receiving CIEDs implantation the clinical course can be complicated by the occurrence of device infection. Rate of infections is usually ≤2%, but such event has an important impact on the clinical management of patients, with high healthcare-associated costs, prolonged hospitalisation and a high rate of mortality events (~20%) [5,6,7,8,9]. It is already known that, in determining the pathophysiological mechanism of the onset of CIEDs infection, various risk factors are involved, both clinical and procedural [10,11,12,13,14,15,16,17,18].

Given the relevant implications of the CIEDs infection, it became pivotal to identify those patients with a higher risk of developing such infection, to implement adequate preventive strategies. To stratify the infective risk, several clinical scores have been proposed, such as those proposed by Shariff and Kolek [19,20,21] and more recently the ‘Prevention of Arrhythmia Device Infection Trial’ (PADIT) score [5].

To provide a contemporary epidemiological assessment of the risk of CIEDs infection and associated clinical outcomes in Italy, we planned and performed a nationwide study about practices, clinical management and outcomes of patients receiving a CIED, irrespective of clinical indication and type of device, the ‘Ricerca sulle Infezioni Associate a ImpiAnto o sostituzione di CIED’ (RI-AIAC) Registry. Additionally, we proposed two novel contemporary scores for the risk stratification of CIEDs infection and clinical outcomes, to be compared to currently used risk scores, as proposed by Shariff, Kolek and the PADIT score [5,19,20,21].

## 2. Methods

**Figure 1:**
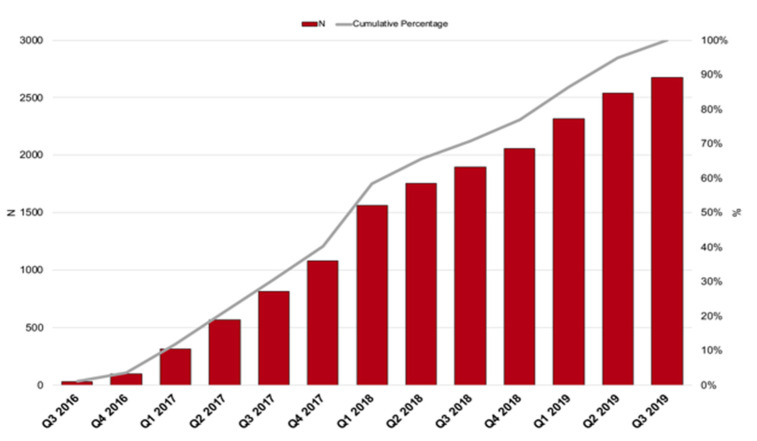
RI-AIAC registry enrolment rates.

The RI-AIAC Registry is an Italian nationwide multicentre prospective observational registry held by the ‘Italian Association of Arrhythmology and Cardiac stimulation’ (Associazione Italiana di Aritmologia e Cardiostimolazione(AIAC)). The registry is based on the AIAC nationwide electrophysiology practices network, promoted by the Cardiology Division at the University of Modena and Reggio Emilia, Policlinico di Modena, which was the coordinating centre. In the first phase of the study, the entire network was claimed for participation to the registry. Forty-three centres answered with an expression of interest. The study was then approved by the Institutional Review Board at AVEN Ethic Committee (Approval N. 9/16, protocol N. 1188/CE) on 01 April 2016. Overall, 18 practices finally entered the study and started the enrolment. Enrolment was undertaken from 1 September 2016 to 31 August 2019, with a progressive and constant accrual rate (Figure 1).

All consecutive patients ≥18 years old undergoing a CIED implant procedure at all sites, irrespective of clinical indication, type of procedure and type of CIED, were approached to obtain informed consent and included in the registry after signing for at least 30 days of enrolment. Baseline data about demographic information, clinical risk factors for CIED infection and procedure main characteristics were collected into an electronic case report form.

All patients enrolled entered a 12-months follow-up observation, with two consecutive visits at 6 and 12 months after the CIED implant procedure. Follow-up visits were performed at each electrophysiology practice. In case a CIED infection was detected, all relevant data were entered into a specific electronic case report form. In the case a death event was detected, date and data regarding the supposed cause of death were recorded separately.

As revision, we defined a procedure where no new prosthetic material was inserted, as upgrade, a procedure where the system characteristics were modified by adding new leads (e.g., from a single chamber ICD to a CRTD system), and as reimplantation the positioning of a new CIED system in a patient who had a previous CIED implant object of extraction.

### 2.1. Outcomes

The outcomes of interest were the occurrence of a CIED infection, as well as the occurrence of any death, irrespective of the cause. In this analysis, we considered the occurrence of CIED infection alone and a composite clinical event of infection or all-cause death.

### 2.2. Statistical Analysis

Continuous variables were reported as mean and standard deviation (SD) or median and interquartile range (IQR) and compared between groups with Independent Samples Student’s T test or Mann–Whitney U test, accordingly. Categorical variables were reported as counts and percentages and compared between groups with Chi-square test.

Incidence of outcomes was calculated by censoring the follow-up time at CIED infection and all-cause death occurrence and considering a total follow-up time of 365 days for each patient which completed the follow-up observation without reporting any event. All patients lost throughout follow-up were not considered for incidence calculation and follow-up analyses.

To establish clinical factors associated with outcomes occurrence, a logistic regression analysis was performed. Clinical factors significantly different at baseline between patients that reported and not reported the outcome were considered for univariate analysis. All factors with a *p* ≤ 0.10 at univariate analysis were included into the multivariate analysis. Results of the logistic regression analysis were reported as odds ratio (OR) and 95% confidence interval (CI).

Based on the coefficients reported at multivariate logistic analysis, we have obtained a clinical score by rounding and re-scaling beta-coefficients, corresponding to the logarithm of the OR estimated during regression procedure. We have elaborated and evaluated two differential scores, one about CIED infection occurrence and one about the composite clinical event occurrence.

The association between the clinical scores considered and the occurrence of the outcomes was evaluated using an adjusted regression analysis. The predictive ability of each score was examined and compared according to De Long and De Long and reported by c-index and 95% CI. A cut-off value for the new scores was established according to the Youden Index.

A two-sided *p* < 0.05 was considered statistically significant. All analyses were performed using SPSS statistical software version 27.0.1.0 (IBM, Armonk, NY, USA) and STATA/MP 16.1 (StataCorp, College Station, TX, USA) for MacOS.

### 2.3. External Validation

We performed an external validation of the two newly proposed scores in an independent cohort of 1017 consecutive patients receiving a CIED implant procedure in a third-level referral electrophysiology centre, collected consecutively from 1 January 2017 to 31 December 2019. We evaluated association between the clinical scores and the outcomes, as well as the predictive performance of each score using the same logistic model and the same methods used in the main analysis. All the patients were followed by the centre’s physicians, using similar procedures to those implemented in our study. Moreover, for this cohort 1 year follow-up data were assessed.

## 3. Results

A total of 2675 patients were enrolled throughout the study period in 18 active sites. Patients were enrolled prevalently in Northern Italy, with 1658 (62%) among 11 sites, while 571 (21.3%) were enrolled in Central Italy (5 centres) and 446 (16.7%) in Southern Italy (2 centres). Baseline characteristics are reported in Table 1. Overall, the study cohort was significantly old, with 1623 (60.7%) patients being ≥75 years old, and prevalently male (1720 patients (64.3%)). Most of the procedures were performed in a ward setting, with a majority of first implantation (70.1%). Regarding the type of device, 1785 (66.7%) patients received a PM, 450 (16.8%) an ICD, 106 (4.0%) a CRT-P and 329 (12.3) a CRT-D. Among the known risk factors for CIEDs infection, heart failure and the use of oral anticoagulants were the most common, being present in 27.7% and 30.7%, respectively.

Overall, our cohort had a median (IQR) of 2 (1,2) infection risk factors, with an overall low risk according to all the scores examined (Table 1).

Antibiotic prophylaxis was performed in almost all the patients enrolled, with cephalosporins being the agent most used. Among the overall cohort, only 323 (12.1%) patients were treated with intra-operatory pocket irrigation with antibiotics. Only 53 patients (2%) received at implant an absorbable antibacterial envelope.

### 3.1. Follow-Up and Incidence of Adverse Outcomes

**Figure 2:**
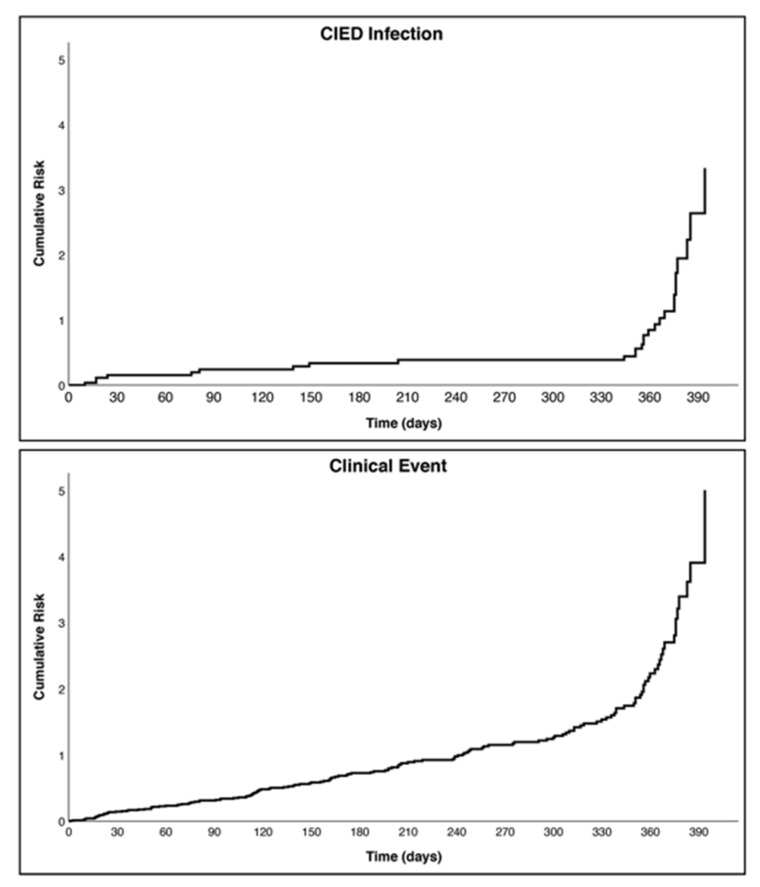
Kaplan–Meier curves for clinical outcomes occurrence. (Upper Figure) and (Cumulative risk for CIED infection; Lower Figure) Cumulative risk for composite clinical event; Legend: CIED = Cardiac Implantable Electronic Device.

Throughout the follow-up observation, a total of 2647 patients (99%) had available data, with 2523 (94.3%) with available data regarding the occurrence of infection, due to censoring of observation. Overall, there were 28 (1.1%) CIED infections and 132 (5%) deaths, with 152 (5.7%) composite clinical events. Cumulative risk of clinical outcomes is reported in Figure 2. Mean (SD) follow-up for CIED infection was 276.25 (143.61) days, while mean (SD) follow-up for composite clinical event was 273.57 (141.65) days.

Overall, the final incidence of CIED infection was equal to 1.1 per 100 patient-years, while the final incidence of the composite clinical event was 5.82 per 100 patient-years.

### 3.2. Clinical Factors Associated to Outcomes Occurrence

According to baseline characteristics, patients developing a CIED infection were younger than those not developing this complication (*p* = 0.041), even though there was no difference according to age classes (*p* = 0.538) (Table 2). Patients presenting a CIED infection had been more likely enrolled for a revision/upgrading/reimplantation with an early revision, receiving less likely a PM (Table 2). Furthermore, patients developing a CIED infection were more likely diabetic, with a severe kidney disease and an hospital-acquired infection (HAI) (Table 2).

According to logistic univariate analysis (Table 3), a multivariate analysis was performed, which identified the type of procedure (revision/upgrading/reimplantation vs. first implantation OR: 4.08, 95% CI: 1.38–12.08) and history of diabetes mellitus (OR: 2.22, 95% CI: 1.02–4.84) as main clinical factors associated to CIED infection occurrence, with the presence of an HAI showing a trend in association (OR: 3.96, 95% CI: 0.85–18.57, *p* = 0.080) (Table 3).

Regarding the occurrence of the composite clinical event, at baseline those patients who developed an event were significantly older, more likely diabetic and with chronic kidney disease even at a severe stage and also were more likely to have experienced a prolonged period of temporary pacing and had presented more often an HAI. After univariate regression analysis, multivariate analysis showed that a progressively increasing age, a prolonged period of temporary pacing, use of oral corticosteroids and the presence of an HAI were significantly associated with the occurrence of a composite clinical event, with diabetes mellitus showing a strong trend in association (Table 4).

### 3.3. Association and Performance of Clinical Risk Scores

Based on the multivariate logistic regression analysis, we firstly developed a score regarding the occurrence of CIED infection, the RI-AIAC Infection score, by scoring 1 point for any CIED replacement, 2 points for revision / upgrading / reimplantation, 1 point for presence of diabetes mellitus and 1 point for reporting a HAI (Table 3).

**Figure 3:**
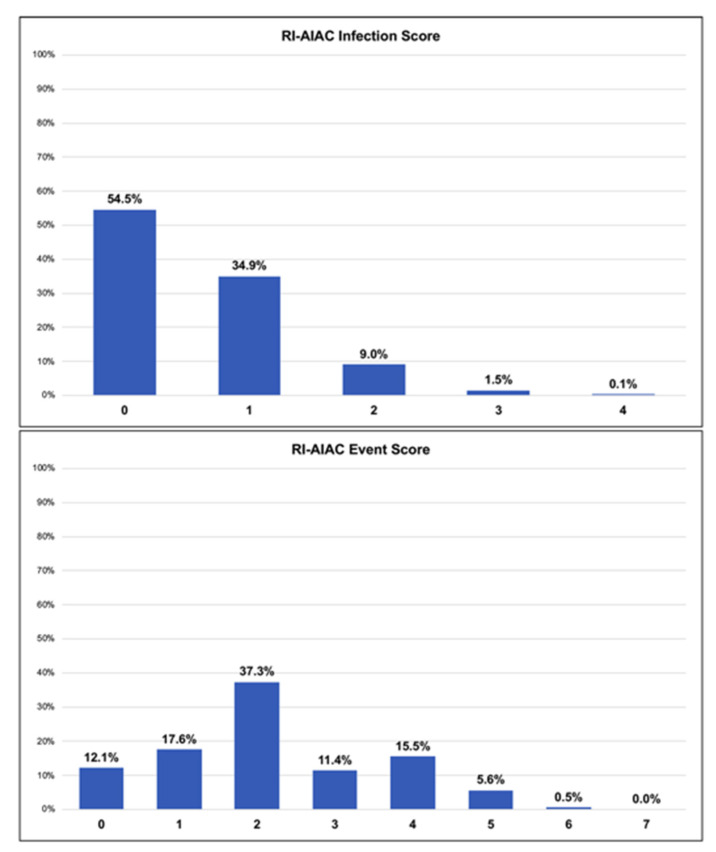
RI-AIAC scores distribution; (Upper Figure RI-AIAC Infection Score; Lower Figure) RI-AIAC Event Score; RI-AIAC = Ricerca sulle Infezioni Associate a ImpiAnto o sostituzione di CIED.

Table 5 itemises simple clinical scores including age classes, presence of delayed temporary pacing, chronic kidney disease, diabetes mellitus and use of oral corticosteroids and HAI (Table 4). Scoring for each item was assigned according to the multivariate logistic regression analysis (Table 4). Distribution of patients according to the two scores is shown in Figure 3.

We tested the association between the pre-existing scores, together with the new proposed ones (Table 6). After adjustments, while Kolek and Shariff scores were not associated with CIED infection occurrence, both the PADIT score and the RI-AIAC Infection score were significantly associated with CIED infections (Table 6), with the RI-AIAC infection score showing the strongest association (OR: 2.38, 95% CI: 1.60–3.55 for each point). Regarding the occurrence of the composite clinical event, while the PADIT score was not associated with the event, the Kolek and Shariff scores were both associated with the event, as well as the RI-AIAC Event score, which showed the strongest association (OR: 1.56, 95% CI: 1.38–1.75) (Table 6).

### 3.4. Predictive Ability of Clinical Scores

The four scores were tested for predictive capacity regarding the two clinical outcomes. For the occurrence of CIED infection, both the PADIT score and the RI-AIAC Infection score showed an overall modest prediction performance, while both Kolek and Shariff scores were not able to predict the outcome (Table 6), with no significant difference between the two scores (*p* = 0.87). Regarding the occurrence of the composite clinical event, Kolek score, Shariff score and RI-AIAC Event score all predicted the outcome occurrence (Table 6)., The RI-AIAC Event score showed a modest to good predictive performance (c-index: 0.67, 95% CI: 0.63–0.71), being superior both to Kolek score (*p* = 0.008) and to Shariff score (*p* = 0.006).

According to the Youden Index, a RI-AIAC Infection score ≥1 identified a patient at higher risk of CIED infection (Youden Index: 0.254, Sensitivity 36%, Specificity 90%). While a RI-AIAC Event score ≥2 (Youden Index: 0.272, Sensitivity 59%, Specificity 69%) identified a patient at higher risk of the composite clinical event. A RI-AIAC Infection score ≥1 was significantly associated with CIED infection occurrence (OR: 2.23, 95% CI: 1.02–4.85), after adjustment for age and sex. RI-AIAC Event score ≥2 was significantly associated with the composite clinical event occurrence (OR: 3.87, 95% CI: 2.29–6.56), after adjustment for sex.

### 3.5. External Validation Analysis

The external validation cohort consisted of 1017 patients, with a median (IQR) age of 83 (74–88) years and 630 (61.9%) male patients. Overall, 750 (73.7%) patients were ≥75 years and 622 (61.2%) patients were ≥80 years. Among the overall cohort, 819 (80.5%) patients received a PM implant, while the remaining 198 (19.5%) received an ICD implant. Median (IQR) PADIT score was 1 (0–3), with a maximum score of 12, median (IQR) Kolek score was 2 (1,2), median (IQR) Shariff score was 2 (1–3), median (IQR) RI-AIAC Infection score was 1 (0,1) and median (IQR) RI-AIAC Event score was 3 (2,3). Accordingly, a RI-AIAC Infection score ≥1 was found in 544 (53.5%) patients and a RI-AIAC Event score ≥2 was found in 866 (85.2%) patients. Throughout the 1-year observation, there were 14 (1.4%) CIED infections, with an overall incidence of 1.48 per 100 patient-years. Moreover, there were 121 (11.9%) composite clinical events, with an overall incidence of 12.72 per 100 patient-years.

Logistic regression analysis, adjusted for age and sex, showed that RI-AIAC Infection score was associated with occurrence of CIED infection (OR: 2.12, 95% CI: 1.09–4.12). Furthermore, the RI-AIAC Event score was associated with occurrence of the composite clinical event (OR: 1.84, 95% CI: 1.51–2.23), after adjustment for sex.

Results of the predictive ability analysis in this external validation cohort were reported in Table 6. Regarding the prediction of CIED infection occurrence, none of the scores resulted able to predict the outcome in this cohort (Table 6). Conversely, regarding the occurrence of the composite clinical event, the Shariff score showed a poor predictivity, while both the Kolek and the RI-AIAC Event scores showed modest to good predictivity (Table 7). Comparing the three scores, while the RI-AIAC Event score showed consistent better predictive ability than the Shariff score (*p* < 0.001), no difference was found between the RI-AIAC Event and the Kolek score (*p* = 0.200). Performing a complete evaluation of the two scores, while the Kolek score showed moderate sensitivity and specificity, the RI-AIAC Event score showed very good sensitivity (95.9%) but very low specificity (16.3%).

## 4. Discussion

In the Italian nationwide RI-AIAC registry, we showed that in a real-life contemporary cohort of patients receiving a CIED implantation the majority of procedures performed refers to PM implantation in an hospital setting. Patients included in the study were generally old, with several prevalent comorbidities and an overall low risk of infection. Throughout follow-up observation, we recorded a 1-year incidence of CIED infections of 1.1%, comparable with some recent prospectively collected data, such as the WRAP-IT trial [22] but lower than the rates of 2.2–2.4% reported in other studies [23,24] or the rate up to 5% reported by some centres in the EHRA survey [25]. Notably, in our study, the risk of CIED infection was higher at longer term from the device implant, with a different occurrence of early (occurring 1–30 days after CIED implant), mid-onset (occurring in the range 31–182 days after implant) and late-onset infections [26,27] with the latter being more common. In our cohort, the antibiotic envelope TYRX, already available at the time of our study initiation, was applied only to few selected cases, and we can hypothesize it was associated with a reduced occurrence of CIED infections in the first months after CIED implant, as shown in the WRAP-IT trial [22].

In this real-world cohort study, we found a high incidence of a composite clinical event of CIED infection or all-cause death. Previous studies that reported a high mortality, up to 20–25% at 1 year, were related to patients associated with CIED infection [28] or to patients with infections undergoing lead extraction in referral centres [29]. This appears to be in contrast with studies on long term outcomes after PM implant, showing a survival of around 55% at 10 years [30] or with the outcome of ICD or CRTD devices, which is characterized by a survival of around 60% at 5 years [31].

Our study derived a score for predicting both the risk of CIED infection and a composite clinical event of infection or all-cause death. We decided to keep separate the two assessments since only some of the independent predictors of the clinical composite end point were independent predictors of infections alone, and the overall incidence of the composite clinical score was more than 5-fold higher than the incidence of infection. The rationale for this choice may also be related to the consideration that age may act on different directions about some outcomes. Indeed, younger age was found to be a risk factor for CIED infections [11] and is actually included in the PADIT score [5]. Although we did not find an independent association between age classes and infections, we found conversely that a more advanced age was associated with the clinical composite outcome. Assessment of all-cause mortality appears necessary when evaluating a contemporary cohort of device patients in view of the important increase in ultra-octogenarian patients treated with PM implant in recent years [4]. Among the factors associated with the occurrence of the clinical outcomes, we found that most of the factors are associated with the presence of relevant comorbidities or with a more deprived clinical status. This evidence is reinforced by the analysis of the external validation cohort, which showed more than 60% of octogenarian patients, reporting a very high incidence of the composite clinical event, being more than 12% over 1 year of observation.

As reported above, previous literature reported a wide range for the incidence of CIED infections, with a trend towards a decline in most recent years [11,23,24,25]. Such trend is strengthened by our data, probably also underlining an improvement in the management of such patients resulting in a lower incidence of infections. Furthermore, even a recent epidemiological analysis underlined how several risk factors exist for CIED infection occurrence, whose risk can be strongly dependent on the cohort examined, being related to device, patient and procedural characteristics [11]. All these aspects likely influenced the predictive abilities of the scores tested to identify those patients at higher risk CIED infection occurrence. On one hand, the Kolek and the Shariff scores were derived from older cohorts with a different risk profile [19,20,21], on the other hand, the PADIT score derived from a clinical trial cohort stemmed from a highly selective cohort [5]. Moreover, the prevalence of PM implants was very low, differently from what can be found in real-life cohorts as those presented in our paper in which PM represents the vast majority. All these specific factors, together with a probably more complex pathophysiology such as that captured by a clinical risk score which is reductionist by definition, could have prevented a good discriminative ability for the previous scores and the proposed RI-AIAC Infection score. Such scenario is also reinforced by other data coming from different medical fields, which showed a large heterogeneity and a substantial failure of almost of all the proposed scores to identify patients at higher risk of developing an infective complication [32].

Contrarily, the occurrence of all-cause death seems to be increasing over time becoming even a more feared complication than the CIED infection itself [30,31]. In this regard, the predictive performance of the RI-AIAC Event score appears to be reassuring, reporting a moderate to good predictive ability both in the derivation and validation cohorts. Furthermore, the very high sensitivity showed underlines the ability to identify all those patients with the highest risk of reporting a significant clinical event, whether an infection or death, to adopt more intensive treatments and a stricter follow-up strategy to minimize the risk. At the same time, even though the Kolek score showed a similar predictive ability, the RI-AIAC Event has a lower number of variables included which are easy to collect bedside, and it is also specifically derived for the composite of major adverse outcomes from a contemporary cohort. Based on such similar predictive ability, the easiness of compilation for the RI-AIAC Event score can help physicians obtaining a simple and rapid risk stratification for patients undergoing a CIED implant in every clinical setting, to identify those patients for which a more intensive and specific management is deemed to reduce the risk of adverse outcomes. On these premises, we believe that the application of a clinical score to predict the occurrence of all the relevant clinical events, such as the RI-AIAC Event score which can provide a good balance between evidence, precision and practicality [33], would be beneficial in the management of these patients.

### Strength and Limitations

In the field, many evaluations on the rate of infections were retrospective or derived from administrative datasets [9], but administrative datasets were found result in not really accurate assessments of infection rates with a high risk of CIED infections underreporting [34]. Our study is original in prospectively investigating a cohort of patients from daily clinical practice and in deriving a score both for infection at 1 year and, separately, for the clinical end point of infection or cardiac death. As recently stressed [11], the large majority of reports on CIED infections were retrospective, and also, the derived scores for predicting the risk of CIED infection were derived from retrospective analysis with the only exception of the PADIT score.

Among the limitations, we have to consider that the diagnosis of CIED infection was based on the diagnosis made by each investigator, as per standard care, without any centralized adjudication. Moreover, data regarding the cause of death were missing, and we could not deepen the analysis regarding the risk factors and the occurrence of death further. Furthermore, as this is an observational study, we cannot exclude the presence of other factors which could have influenced the occurrence of the clinical outcomes considered. Moreover, specific data regarding the occurrence of infective endocarditis were not collected in the study. Notwithstanding, data presented in this paper reflect the “real world” characterization of the present study, including a substantial amount of pacemaker implants and of CIED replacements [35]. Conversely, our analysis being limited to Italy, generalization of these results needs to be made with caution.

## 5. Conclusions

In this Italian nationwide cohort of patients receiving a CIED implant, we found that while incidence of CIED infections was substantially low, and the rate of the composite clinical outcome of infection or all-cause death was quite high and substantially associated with several clinical factors depicting a more impaired clinical status. A simple clinical score derived from those factors, the RI-AIAC Event score, has been found to be significantly associated with the occurrence of the composite clinical outcome, showing a modest to good predictive ability, also confirmed in the external validation cohort. Application of the RI-AIAC Event score would help to identify those patients at higher risk of reporting an adverse clinical event and adopt specific strategies to minimize this risk.

## Figures and Tables

**Table 1:** Baseline characteristics of enrolled patients.

	*N* = 2675
**Age**, years median (IQR)	78 (70–84)
**Age Classes**, *n* (%) <65 years 65–74 years ≥75 years	422 (15.8) 630 (23.6) 1623 (60.7)
**Male Sex**, *n* (%)	1720 (64.3)
**Admission**, *n* (%) Ward Daily Service	2263 (84.6) 412 (15.4)
**Procedure**, *n* (%) First Implantation Replacement Further Replacement Contralateral Implantation Upgrading Revision Other	1874 (70.1) 448 (16.7) 205 (7.7) 17 (0.6) 72 (2.7) 51 (1.9) 8 (0.3)
**CIED Type**, *n* (%) Pacemaker ICD CRT-P CRT-D Other	1785 (66.7) 450 (16.8) 106 (4.0) 329 (12.3) 5 (0.2)
**>2 Leads**, *n* (%)	194 (7.3)
**Early Revision**, *n* (%)	38 (1.4)
**Prolonged Temporary Pacing**, *n* (%)	73 (2.7)
**eGFR <60 mL/min**, *n* (%)	622 (23.3)
**Pre-Dialysis/Dialysis**, *n* (%)	41 (1.5)
**Diabetes Mellitus**, *n* (%)	550 (20.6)
**Heart Failure**, *n* (%)	740 (27.7)
**Use of Oral Corticosteroids**, *n* (%)	72 (2.7)
**Use of Oral Anticoagulants**, *n* (%)	821 (30.7)
**Use of Immunosuppressive Therapy**, *n* (%)	18 (0.7)
**24 h Pre-Implantation Fever**, *n* (%)	12 (0.4)
**Hospital-Acquired Infection**, *n* (%)	44 (1.6)
**Antibiotic Prophylaxis**, *n* (%)	2636 (98.5)
**Antibiotic Type**, *n* (%) 2636 Cephalosporins Clindamycin Penicillin Other	2186 (82.9) 70 (2.6) 250 (9.3) 130 (4.9)
**Overall Infection Risk Factors**, *n* median [IQR]	2 (1,2)
**PADIT Score**, median [IQR]	1 (0–4)
**Kolek Score**, median [IQR]	1 (0–2)
**Shariff Score**, median [IQR]	1 (0–2)

CIED = Cardiac Implantable Electronic Device; CRT-D = Cardiac Resynchronization Therapy Defibrillator; CRT-P = Cardiac Resynchronization Therapy Pacing; eGFR = Estimated Glomerular Filtration Rate; ICD = Implantable Cardioverter Defibrillator; IQR = Interquartile Range; PADIT = Prevention of Arrhythmia Device Infection Trial; SD = Standard Deviation.

**Table 2:** Baseline characteristics according to Occurrence of Infection during Follow-Up.

	No Infection *N*= 2495	Infection *N*= 28	*p*
**Age**, years median (IQR)	77 (69–83)	74 (66–77)	0.041
**Age Classes**, *n* (%) <65 years 65–74 years ≥75 years	411 (16.5) 597 (23.9) 1487 (59.6)	5 (17.9) 9 (32.1) 14 (50)	0.538
**Male Sex**, *n* (%)	1601 (64.2)	19 (67.9)	0.686
**Admission**, *n* (%) Ward Daily Service	2112 (84.6) 383 (15.4)	23 (82.1) 5 (17.9)	0.715
**Procedure**, *n* (%) First Implantation Replacement Further Replacement Contralateral Implantation Upgrading Revision Other	1745 (69.9) 428 (17.2) 186 (7.5) 14 (0.6) 67 (2.7) 48 (1.9) 7 (0.3)	15 (53.6) 2 (7.1) 4 (14.3) 3 (10.7) 2 (7.1) 1 (3.6) 1 (3.6)	<0.001
**CIED Type**, *n* (%) Pacemaker ICD CRT-P CRT-D Other	1654 (66.3) 426 (17.1) 98 (3.9) 312 (12.5) 5 (0.2)	13 (46.5) 8 (28.6) 3 (10.7) 4 (14.3) 0	0.131
**CIED Type Recode**, *n* (%) Pacemaker Any Other CIED	1654 (66.3) 841 (33.7)	13 (46.4) 15 (53.6)	0.027
**>2 Leads**, *n* (%)	184 (7.4)	3 (10.7)	0.502
**Early Revision**, *n* (%)	34 (1.4)	2 (7.1)	0.010
**Prolonged Temporary Pacing**, *n* (%)	62 (2.5)	1 (3.6)	0.714
**eGFR <60 mL/min**, *n* (%)	553 (22.2)	6 (21.4)	0.926
**Pre-Dialysis/Dialysis**, *n* (%)	32 (1.3)	2 (7.1)	0.007
**Diabetes Mellitus**, *n* (%)	499 (20.0)	11 (39.3)	0.012
**Heart Failure**, *n* (%)	689 (27.6)	11 (39.3)	0.170
**Use of Oral Corticosteroids**, *n* (%)	62 (2.5)	1 (3.6)	0.714
**Use of Oral Anticoagulants**, *n* (%)	759 (30.4)	9 (32.1)	0.844
**Use of Immunosuppressive Therapy**, *n* (%)	17 (0.7)	0	1.000
**24 h Pre-Implantation Fever**, *n* (%)	10 (0.4)	0	1.000
**Hospital-Acquired Infection**, *n* (%)	37 (1.5)	2 (7.1)	0.016
**Antibiotic Prophylaxis**, *n* (%)	2455 (98.4)	28 (100)	0.499
**Antibiotic Type**, *n* (%) 2636 **Cephalosporins** Clindamycin Penicillin Other	2055 (83.7) 61 (2.5) 224 (9.1) 116 (4.7)	19 (67.9) 2 (7.1) 5 (17.9) 2 (7.1)	0.120
**Overall Infection Risk Factors**, n median [IQR]	2 (1–2)	2 (1–3)	0.086
**PADIT Score**, median [IQR]	1 (0–4)	4 (1–5)	0.008
**Kolek Score**, median [IQR]	1 (0–2)	1 (0–2)	0.136
**Shariff Score**, median [IQR]	1 (0–2)	2 (1–3)	0.080

CIED = Cardiac Implantable Electronic Device; CRT-D = Cardiac Resynchronization Therapy Defibrillator; CRT-P = Cardiac Resynchronization Therapy Pacing; eGFR = Estimated Glomerular Filtration Rate; ICD = Implantable Cardioverter Defibrillator; IQR = Interquartile Range; PADIT = Prevention of Arrhythmia Device Infection Trial; SD = Standard Deviation.

**Table 3:** Logistic regression analysis for CIED infection occurrence.

	Univariate Analysis		Multivariate Analysis		Score Point
	OR (95% CI)	*p*	OR (95% CI)	*p*	
**Age Classes** <65 years (ref.) 65–74 years ≥75 years	- 1.24 (0.41–3.72) 0.77 (0.28–2.16)	- 0.702 0.625	- - -	- - -	- - -
**Procedure** First Implantation Any Replacement Revision/Upgrading/Reimplantation	- 1.14 (0.44–2.94) 5.99 (2.4–14.94)	- 0.792 <0.001	- 1.21 (0.46–3.18) 4.08 (1.38–12.08)	- 0.698 0.011	0 1 2
**CIED** Pacemaker (ref.) Any Other CIED	- 2.27 (1.08–4.79)	- 0.032	- 1.66 (0.75–3.68)	- 0.209	- -
**Early Revision**	5.57 (1.27–24.40)	0.023	1.68 (0.30–8.87)	0.579	-
**Pre-Dialysis/Dialysis**	5.92 (1.35–26)	0.019	3.44 (0.74–15.94)	0.116	-
**Diabetes Mellitus**	2.59 (1.21–5.56)	0.015	2.22 (1.02–4.84)	0.045	1
**Hospital-Acquired Infection**	5.11 (1.17–22.32)	0.030	3.96 (0.85–18.57)	0.080	1

CI = Confidence Interval; CIED = Cardiac Implantable Electronic Device; OR = Odds Ratio.

**Table 4:** Logistic regression analysis for clinical events occurrence.

	Univariate Analysis		Multivariate Analysis		Score Points
	OR (95% CI)	*p*	OR (95% CI)	*p*	
**Age Classes** <65 years (ref.) 65-74 years ≥75 years	- 2.32 (1.05–5.17) 4.04 (1.96–8.34)	- 0.039 <0.001	- 2.07 (0.92–4.65) 3.10 (1.45–6.64)	- 0.079 0.006	0 1 2
**CIED** Pacemaker (ref) Any Other CIED	- 0.7 (0.49–1.02)	- 0.062	- 0.95 (0.64–1.41)	- 0.798	
**Prolonged Temporary Pacing**	3.06 (1.58–5.94)	0.001	2.9 (1.45–5.77)	0.002	1
**eGFR <60 mL/min**	2.55 (1.83–3.57)	<0.001	2.03 (1.43–2.88)	<0.001	1
**Pre-Dialysis/Dialysis**	1.62 (1.25–2.11)	<0.001			
**Diabetes Mellitus**	1.58 (1.09–2.28)	0.015	1.43 (0.98–2.08)	0.063	1
**Use of Oral Corticosteroids**	2.47 (1.2–5.08)	0.014	2.60 (1.25–5.40)	0.010	1
**Hospital-Acquired Infection**	3.21 (1.41–7.32)	0.006	3.29 (1.29–8.35)	0.012	1

CI = Confidence Interval; CIED = Cardiac Implantable Electronic Device; eGFR = Estimated Glomerular Filtration Rate; OR = Odds Ratio.

**Table 5:** Predictive ability of clinical scores.

	Infection		Clinical Events	
	C-Index (95% CI)	*p*	C-Index (95% CI)	*p*
**PADIT Score**	0.64 (0.53–0.76)	0.010	0.51 (0.47–0.56)	0.612
**Kolek Score**	0.56 (0.46–0.66)	0.261	0.63 (0.59–0.68)	<0.001
**Shariff Score**	0.58 (0.47–0.68)	0.159	0.62 (0.58–0.67)	<0.001
**RI-AIAC Infection**	0.64 (0.52–0.75)	0.015	-	-
RI-AIAC Event *	-	-	0.67 (0.63–0.71)	<0.001

* Adjusted for sex; † adjusted for age and sex; CI= Confidence Interval; OR= Odds Ratio; PADIT = Prevention of Arrhythmia Device Infection Trial; RI-AIAC = Ricerca sulle Infezioni Associate a ImpiAnto o sostituzione di CIED.

**Table 6:** Logistic regression of clinical scores.

	Infection		Clinical Events	
	OR (95% CI)	*p*	OR (95% CI)	*p*
PADIT Score *	1.28 (1.1–1.50)	0.002	1.01 (0.94–1.09)	0.748
**Kolek Score †**	1.55 (0.73–3.27)	0.251	1.36 (1.2–1.55)	<0.001
**Shariff Score †**	1.69 (0.89–3.21)	0.112	1.32 (1.17–1.47)	<0.001
**RI-AIAC Infection †**	2.38 (1.6–3.55)	<0.001	-	-
RI-AIAC Event *	-	-	1.56 (1.38-1.75)	<0.001

* Adjusted for sex; † adjusted for age and sex; CI = Confidence Interval; OR = Odds Ratio; PADIT = Prevention of Arrhythmia Device Infection Trial; RI-AIAC = Ricerca sulle Infezioni Associate a ImpiAnto o sostituzione di CIED.

**Table 7:** Predictive ability of clinical scores in external validation cohort.

	Infection		Clinical Events		SE	SP
	C-Index (95% CI)	*p*	C-Index (95% CI)	*p*		
**PADIT Score**	0.53 (0.38–0.67)	0.746	0.49 (0.44–0.53)	0.600	-	-
**Kolek Score**	0.64 (0.5–0.79)	0.065	0.65 (0.6–0.7) *	<0.001	70.3%	51.2%
**Shariff Score**	0.62 (0.46–0.77)	0.131	0.56 (0.51–0.62) ^†^	0.025	-	-
**RI-AIAC Infection**	0.58 (0.42–0.74)	0.292	-	-	-	-
**RI-AIAC Event**	-	-	0.68 (0.63–0.72)	<0.001	95.9%	16.3%

* No significant difference compared to RI-AIAC Event (De Long–De Long *p* = 0.2; ^†^ significantly less predictive than RI-AIAC Event (De Long–De Long *p* < 0.001); CI = Confidence Interval; PADIT = Prevention of Arrhythmia Device Infection Trial; RI-AIAC = Ricerca sulle Infezioni Associate a ImpiAnto o sostituzione di CIED; SE = Sensitivity; SP = Specificity.

## Data Availability

Data will be provided upon reasonable request.
